# Internal Secretions and Their Functions

**Published:** 1916-06-17

**Authors:** 


### INTERNAL SECRETIONS AND THEIR FUNCTIONS.

### A New Chapter in Modern Physiology.

The term a "secretion" is one familiar to every student of physiology. Until recent years it meant a substance formed in a pouch or gland and passed from the gland by a tube or duct on to a free surface. Thus numberless minute glands open on the surface of the skin and secrete the sweat; larger and more complicated pouches secrete the saliva which flows on to the lining membrane of the mouth; and in similar fashion the gastric juice is formed by the glands of the stomach. In short, alike on the free surface of the skin and of every mucous membrane are the openings of glands through which pass secretions formed in the epithelial pouches or recesses of which the glands are composed. Such secretions have the form of watery fluids, but they contain, at least for the most part, active principles which render some form of service to the body. For example, the saliva contains *ptyalin* which assists in the digestion of starchy foods, while the *pepsin* of the gastric juice is the digestive agent for proteids. To this point, therefore, the phrase a "secretion" implies the existence of a gland provided with a duct through which the materials formed by the gland reach an external or free surface. Such was the physiological position to which the student was introduced up to the end of the nineteenth century. It is true that he was told also of certain organs which were named collectively "ductless glands," but what part these played in the economy was left an entirely open question, and indeed some of these structures were regarded as effete or useless. Still the use of the word "gland" in this connection did express in a vague fashion the notion that these organs "formed" something, but what this something might be or what value it might possess were unsolved puzzles.

Now it is from the acquisition of knowledge relative to the function of certain of these despised "ductless glands" that the term "internal secretion" has come into use. Instead of being of uncertain or doubtful value these organs are now known to play a highly important part in the smooth working of the bodily machinery. And they play this part by the formation within themselves of certain chemical agents. The process may therefore be compared to that which goes on in an ordinary gland, and hence, not unnaturally, it is spoken of as "secretion." But the substances so formed do not pass through a duct on to an external or free surface. On the contrary, they pass from the organs in which they are produced into the blood stream. Hence it has come about that these substances are termed "internal" secretions. In a word, while ordinary or external secretions are formed in recesses or glands, and pass through the duct of the gland on to a free surface, internal secretions are produced in organs which have no duct, and the secretion is contributed to the circulating blood.

There are reasons for believing that many of the organs and tissues of the body make contributions to the blood in the fashion just described—that is, internal secretion is a function probably widely spread in the body. But precise knowledge is at present limited mainly to certain organs which, so far as is known, have no function other than the formation of internal secretions. These are the thyroid, the para-thyroids, the adrenals, and the pituitary body. Of the nature of the internal secretion yielded by each of these we possess nowadays a very confident knowledge, as also of the part which each such secretion plays in the economy. The development is an enormous one, for the student of some twenty years ago regarded the value of each of these organs as an impenetrable secret and probably heard about them no more helpful suggestion than an ancient speculation which proposed the pituitary body as the seat of the soul!

What is the particular action of each "internal secretion," or *autacoid*, in the organism is a long and complicated story, and no attempt to present such a story can be made here. But it may be said generally that each of these agents has an effect on one or more of the organs of the body, and that this effect is in the shape of a modification of the activity of the organ or organs concerned. Such modification may be in the direction of increase or in the direction of diminution. That is to say, there are some autacoids which have a stimulating effect on certain bodily activities, and these it is proposed to call *hormones*; there are others which exercise a restraining or inhibiting effect on various functions, and these are spoken of as *chalones*. Presumably, therefore, what may be called the normal or healthy level of functional activity in the body depends on the nice adjustment of these substances in the blood, and this in turn depends on the satisfactory working of the organs in which the autacoids are produced. If any one of these organs is not working satisfactorily trouble may be expected. The organ may conceivably either fail to produce a proper amount of its internal secretion or it may produce this in excess; and corresponding to the one and the other there will be a disturbance in the working of the bodily machine. The practical medical application of these physiological advances arises from the fact that we are gradually learning to identify the particular disturbance which defect or excess of each of the internal secretions produces; and from this there springs a method of treatment. For example, it is known that the disease called myxœdema depends upon a defective supply of the internal secretion of the thyroid, and that when the patient receives thyroid secretion, as obtained from the sheep or other animal, the symptoms disappear. In other directions the matter is not so simple, and there is still much to be learned; but it is certain that the study of the internal secretions has opened a chapter as important to the physician as it is fascinating to the physiologist.

